# Non-Invasive *In Vivo* Imaging of Near Infrared-labeled Transferrin in Breast Cancer Cells and Tumors Using Fluorescence Lifetime FRET

**DOI:** 10.1371/journal.pone.0080269

**Published:** 2013-11-21

**Authors:** Ken Abe, Lingling Zhao, Ammasi Periasamy, Xavier Intes, Margarida Barroso

**Affiliations:** 1 Albany Medical College, The Center for Cardiovascular Sciences, Albany, New York, United States of America; 2 Rensselaer Polytechnic Institute, Department of Biomedical Engineering, Jonsson Engineering Center Troy, New York, United States of America; 3 W. M. Keck Center for Cellular Imaging, University of Virginia, Charlottesville, Virginia, United States of America; University of Pécs Medical School, Hungary

## Abstract

The conjugation of anti-cancer drugs to endogenous ligands has proven to be an effective strategy to enhance their pharmacological selectivity and delivery towards neoplasic tissues. Since cell proliferation has a strong requirement for iron, cancer cells express high levels of transferrin receptors (TfnR), making its ligand, transferrin (Tfn), of great interest as a delivery agent for therapeutics. However, a critical gap exists in the ability to non-invasively determine whether drugs conjugated to Tfn are internalized into target cells *in vivo*. Due to the enhanced permeability and retention (EPR) effect, it remains unknown whether these Tfn-conjugated drugs are specifically internalized into cancer cells or are localized non-specifically as a result of a generalized accumulation of macromolecules near tumors. By exploiting the dimeric nature of the TfnR that binds two molecules of Tfn in close proximity, we utilized a Förster Resonance Energy Transfer (FRET) based technique that can discriminate bound and internalized Tfn from free, soluble Tfn. In order to non-invasively visualize intracellular amounts of Tfn in tumors through live animal tissues, we developed a novel near infrared (NIR) fluorescence lifetime FRET imaging technique that uses an active wide-field time gated illumination platform. In summary, we report that the NIR fluorescence lifetime FRET technique is capable of non-invasively detecting bound and internalized forms of Tfn in cancer cells and tumors within a live small animal model, and that our results are quantitatively consistent when compared to well-established intensity-based FRET microscopy methods used in *in vitro* experiments.

## Introduction

A major challenge in optimizing drug delivery is the ability to non-invasively monitor and quantify drug internalization into targets within live subjects. This is especially difficult in diseases such as cancer due to the enhanced permeability and retention (EPR) effect that results in the accumulation of macromolecules in the region of the tumor caused by increased tumor vascular permeability and decreased lymphatic drainage [1], [2]. While this non-specific mechanism of accumulation has been extensively exploited to deliver anti-cancer drugs in the vicinity of diseased tissues, the actual amount of drugs internalized into target tumor cells remains unknown without the use of invasive biochemical studies [3]. Another mechanism to target anti-cancer drugs to a variety of tumors *in vivo* relies on the use of drugs conjugated to native ligands or antibodies that bind the extracellular domain of membrane-bound receptors enriched in tumor tissues [4]. However, the inability to distinguish soluble labeled antibodies or ligands present in the blood and other tissues from those internalized into tumors remains a significant hurdle in their optimization as an efficient drug delivery strategy [5]–[8].

Therefore, in diagnostic and therapeutic cancer research, there is a critical need to develop a non-invasive imaging approach to determine whether candidate ligands or probes are internalized into tumors. Variations in cellular uptake are likely to indicate the usefulness and effectiveness of the drug therapy. Although different imaging techniques such as PET and SPECT have been used to measure radiolabeled probe accumulation in tumors vs. other organs, various factors can affect the accuracy of these measurements. Examples include the label lifetime and the effect of labeling procedures on probe tumor binding affinity, detection sensitivity and specificity, and relative contributions of blood and tissue to determine signal to noise level of tumor labeling [7]. Recently, ^89^Zr was shown to be a very efficient labeling reagent for imaging tissue distribution in cancer therapeutics and diagnostics [9]. However, a common pitfall of PET imaging studies lies in their inability to non-invasively measure specific tumor labeling at earlier time points post injection due to high blood-pool activity, as only *ex vivo* biodistribution studies allow for the measurement of intratumoral peak probe uptake. Moreover, PET *in vivo* imaging at later time points cannot distinguish between specific receptor mediated uptake and unspecific tumor accumulation due to the EPR effect [10]. In this report, we specifically address these limitations by validating a novel, non-invasive, highly sensitive, and quantitative *in vivo* imaging approach that permits the determination of intracellular amounts of internalized receptor-bound ligands used in targeted delivery systems in live animals.

The transferrin receptor (TfnR) has been used extensively as a drug delivery system, since it binds and internalizes iron-bound transferrin (Tfn) to deliver iron into cells [11], [12] Given that the TfnR is over-expressed in tumors compared to normal cells [12], Tfn has been used as a carrier for anti-cancer drugs or other therapeutic agents to enhance targeting specificity towards neoplasic tissues [3], [12], [13]. Likewise, Tfn has also been used in tumor bioimaging upon labeling with radioactive or fluorescent probes, with convincing evidence showing preferential accumulation of Tfn in tumors compared to other non-neoplasic tissues and cells [14]–[20]. Recently, animals carrying xenograft tumors were injected with ^89^Zr-Tfn and PET and biodistribution studies were conducted at multiple time points after injection. These PET studies showed a peak intratumoral uptake at 4 h; however, intratumoral uptake of ^89^Zr-Tfn only exceeded blood-pool activity at 24 h post injection [21], preventing non-invasive measurements of ^89^Zr-Tfn tumor uptake using PET imaging at early time points. Furthermore, this approach intrinsically lacks the ability to distinguish bound and internalized Tfn from unbound, soluble forms of Tfn and therefore cannot distinguish receptor-mediated intracellular Tfn from extracellular soluble Tfn accumulating in the tumor region or in the blood. To accomplish our goal of visualizing and quantifying the receptor-mediated uptake of labeled-Tfn into cancer cells orthotopically implanted into live small animal models, we had to overcome two major challenges. First, we had to develop an approach that could discriminate between soluble and receptor-bound internalized forms of Tfn. We capitalized on the homodimeric nature of the TfnR that binds two molecules of Tfn within 2–10 nm to allow the use of Förster Resonance Energy Transfer (FRET) based imaging techniques [22]. By detecting FRET between appropriately labeled Tfn molecules, we were able to quantitatively determine whether the Tfn is in its bound state at either the plasma membrane and along the endocytic pathway (FRET positive signals), or in its soluble unbound form (FRET negative signals) [23]–[27]. Therefore, this approach allowed us to quantitatively measure the relative amount of intracellular Tfn-TfnR complexe*s* taken up by breast cancer cells. It is important to mention that this approach can be generalized to other antibody based systems that target receptor dimers/oligomers.

The second major challenge was to non-invasively quantify these FRET signals through living tissues in small animal models. Traditional microscopic techniques using visible organic dyes or fluorescent proteins have limited *in vivo* applications based on their high degree of autofluorescence and poor signal penetration depth through biologically heterogeneous tissues [28], [29]. For example, fluorescence lifetime based detection methods have been used in the macroscopic imaging regime to determine FRET between genetically expressed fluorescent proteins (eGFP-mCherry donor-acceptor pair) in the limb of live mice [30]. However, this method requires endogenously expressed fluorescent protein, and is also limited to interrogating small volumes with respect to its detection depth due the visible spectrum range. More recently, self-luminescing BRET-FRET near infrared (NIR) nanoparticles have been used to image lymphatic networks and vasculature of xenografted tumors in living mice [31], nevertheless this method still cannot discriminate between active uptake and non-specific, regional accumulation of particles due to the EPR effect. To address these limitations, we have identified and characterized a fluorophore pair AlexaFluor700 (AF700) and AlexaFluor 750 (AF750) in the NIR range with sufficient brightness, stability, lifetime contrast, while also being capable of achieving a high efficiency of FRET due to a strong R_0_ Förster distance [32]. This NIR FRET pair operates in a spectral window that exhibits low tissue attenuation and minimum tissue auto-fluorescence, overcoming the limited penetration depths using visible FRET pairs. Furthermore, as an alternative to visible and intensity-based FRET microscopy, we developed a NIR fluorescence lifetime FRET macroscope system that detects FRET based on the reduction of donor fluorophore lifetime [33].

This is the first report to use NIR fluorescence lifetime FRET to non-invasively detect Tfn internalization in human cancer cells through living animal tissues. To obtain lifetime data of the donor fluorophore species in the whole animal body and for deep seated tissues, we use a time-resolved, wide-field imaging platform to image small animals in transmission geometry. In turn, the measured fluorescent decays are fit into a bi-exponential decay model that estimates the relative abundances and lifetimes of the donor species, whereby the donor species is assumed to exist in either a FRET or a non-FRET state (i.e., either as a quenched donor or a unquenched donor) [32]. This approach offers a number of advantages including: only measuring the fluorophore lifetime of the donor species for fast acquisition times, providing accurate measurements due to the independence of lifetime contrast from fluorophore concentration, and being robust as fluorophore lifetime measurements are less affected by the heterogeneous nature of the living tissue compared to intensity-based data sets [34]. Since the reduction of lifetime occurs when Tfn binds TfnR and subsequently when Tfn-TfnR complexes are internalized, we can distinguish and quantify unbound Tfn from bound and intracellular Tfn. Thus, this approach provides an analytical method to non-invasively quantify drug internalization in an EPR prone environment with high tumor-blood ratios.

Since fluorescence lifetime FRET requires the binding of Tfn to its receptor to allow donor-labeled Tfn to transfer energy to acceptor-labeled Tfn [35], this imaging approach is able to discriminate between soluble, unbound Tfn localized to blood, tumor or other organs and receptor-bound Tfn internalized specifically into tumor cells. Moreover, we have established that the *in vivo* NIR fluorescence lifetime FRET approach is as quantitatively reliable as data obtained from well-established intensity-based FRET confocal microscopy using *in vitro* samples [23], [24]. These findings clearly demonstrate the feasibility of achieving quantitative, non-invasive detection of internalized NIR-labeled Tfn within live animals to accelerate drug optimization and therapeutic strategies.

## Materials and Methods

### Ethics Statement

All animal protocols were conducted with approval by the Institutional Animal Care and Use Committee at both Albany Medical College and Rensselaer Polytechnic Institute. Minimal discomfort to mice used in this study was ensured using guidelines in accordance with IACUC standards.

### Cell Culture Conditions

T47D (HTB-122) and BT549 (HTB133) cells were purchased from ATCC and cultured in RPMI-1640 media (30–2001, ATCC) as recommended by ATCC. The media formulation was supplemented with 50 units/mL penicillin and 50 µg/mL streptocycin from MP Biomedicals (091670249), and with 10% fetal bovine serum (30–2020 ATCC). Human mammary epithelial cells (HMECs, CC-2551) were purchased from Lonza, using MEGM Complete media (CC-3051) as recommended. All cells were grown in 5% CO_2_ at 37°C in a humidified incubation chamber, and were kept for less than 12 population doublings.

### Tfn Uptake and Confocal Imaging

HMECs and T47D cells were passaged onto MatTek glass bottom culture dishes (P35GC-1.5-14-C) and allowed to grow to ∼75% confluency. For Tfn uptake experiments, cells were washed in phosphate buffered saline solution (PBS) and incubated in Tfn-free imaging media consisting of phenol-free Dulbecco’s Modified Eagle Medium (DMEM) (LifeTechnologies, 31053) and supplemented with 0.37 mg/mL sodium bicarbonate (Sigma, S7277), 5 mg/mL bovine serum albumin (Sigma, A9085), and 4.7 mg/mL HEPES (Sigma, H4034), at 37°C for 1 hour to clear internalized endogenous Tfn. After this pre-incubation period, cells were incubated at 37°C with human Tfn conjugated with AF488 dye purchased from LifeTechnologies, (T13342; ∼2 fluorophore molecules per Tfn molecule) at 100 µg/mL for 0, 1, 2, 5, 10, 20, and 40 minutes. Uptake times were terminated by washing cell chambers with ice-cold PBS followed by cell fixation using 4% formaldehyde for 15 minutes and mounted onto coverslips with DAPI mounting media (LifeTechnologies, P36931). Cells were imaged with a Zeiss Laser Scanning Microscope 510, using a Plan-Apochromat 63x/1.4 oil DIC objective at 488 nm excitation wavelength, followed by detection using a 435–485 nm IR bandpass filter (pinhole = 1000 µm). DAPI imaging was achieved using two-photon excitation at 740 nm, followed by detection using a 500–550 nm IR bandpass filter (pinhole = 98 µm). For FRET analysis using confocal microscopy, cells were incubated for 1 hour at 37°C with media containing donor only (AF488-Tfn), acceptor only (AF555-Tfn) or both at a molar ratio of 2 acceptors to 1 donor molecules. No DAPI was used in FRET experiments. AF700 (LifeTechnologies; A20010) and AF750 (LifeTechnologies, A20011) NIR dye pairs were conjugated onto human Tfn (T3309, Sigma). Firstly, Tfn was saturated with iron after incubation with a molar excess of ferrous ammonium sulfate and nitrilotriacetate sodium salt solution in water. Then, after saturation, Tfn is conjugated to AF700 or AF750 dyes by incubating 5 µl of 10 µg/µl of dye in DMSO per 1 mg of Tfn at a concentration of 7 mg/ml solution. After 1 hour of gentle rotation in the dark, labeling was stopped by passage over a resin desalting column. Tfn-dye complexes were then concentrated using concentrators that exclude the flow-through of particles larger than 35 kD. Samples showed an average degree of labeling of ∼2 fluorophore molecules/Tfn molecule.

### Intensity based FRET Microscopy

Images were acquired on a Zeiss LSM510 META-NLO laser scanning microscope using AIM software (Zeiss USA, Thornwood, NY) configured to donor excitation/donor emission (donor channel), acceptor excitation/acceptor emission (acceptor channel) and donor excitation/acceptor emission (FRET channel). Images were collected at 512×512 pixels, 8-bit depth, and mean of 2 images and at 1x. PMT gain and black-level settings, laser power, and pinhole were set at identical levels for all image collections. Single-labeled images were used to correct Spectral Bleed-Through (SBT) signal contamination.

Precision FRET (PFRET) quantitative analysis algorithm [36] was used to calculate the FRET energy transfer efficiency (E%). After image background-subtraction and processing by the PFRET software, corrected FRET images (PFRET images) are generated for quantitative analyses, as described previously [35].

Energy transfer efficiency, E%, is calculated as the percentage of energy transfer in relation to the unquenched donor, i.e., unquenched donor (D) = quenched donor (qD)+γ⋅PFRET as described in the equation E% = 100×(γ⋅PFRET/D) or E% = 100×[1–(qD/D)]. γ value is a function of the quantum yield of the fluorophores and of the detection setup, where it is assumed to equal 1 as all imaging conditions remained constant [23], [24].

Mathematical models previously described were used to discriminate a clustered from a random membrane protein organization based on the relationship between E% and A levels at specific ranges of acceptor to donor (A:D) ratios [23], [24], [37]–[41]. In a random situation, acceptor co-localization with a donor is positively correlated with acceptor levels and leads to an increase in E%. Conversely, in a clustered situation, E% is largely independent of acceptor levels, and it does not decrease to zero when acceptor trends to zero [38], [39], [42].

### Fluorescence Lifetime Imaging

FRET imaging was performed on a time-domain fluorescence lifetime imaging system using a gated intensified CCD (ICCD) with wide-field illumination described in detail [32]. Instead of a picoprojector, a digital micromirror device (DMD, DLP Discovery 4100 Kit and D2D module, Texas instruments Inc.) is used as a spatial light modulator to create a spatial distribution of light beams by providing a resolution of 1024×768 pixels and 256 grayscale levels. Briefly, a tunable Ti-Sapphire laser (Mai Tai HP, Spectra-Physics, CA, USA) is used as an illumination source to generate 100 fs pulses at 80 MHz in the NIR spectral band (690 nm–1020 nm). The overall laser power is adjustable using a computer-controlled variable attenuator (Application Note 30, Newport) based on the combination of a half-wave plate and a polarizer with a closed loop feedback-control system. The laser beam exiting the power control assembly is coupled into a 400 µm multimode fiber (NA = 0.22), and then it is collimated and focused into the integrator rod of the optical module (S2+optics, Texas instruments Inc.) for projecting the uniform illumination to the imaging stage over 3.2 cm×2.5 cm (up to 8 cm×4 cm). The transmitted light through an emission bandpass filter (FF01-720/13-25 for AF 700, Semrock ) is via a a Nikon 50 mm f/1.8D AF Nikon Lens and detected by the ICCD (PicoStar HR, LaVision GmbH, Germany) allowing the measurement of a maximum of 2^12^ photons with spatial resolution of 1376×1040 pixels). Imaging in all animals and well-plates was performed in transmission geometry.

The instrument response function (IRF) was measured by directly imaging on a white diffusing paper placed on the stage. For all experiments described herein, the excitation wavelength was set at 695 nm and the emission wavelength was set at 720 nm. Furthermore, the gatewidth on the ICCD camera was set to 300 ps. The IRF and the excitation signals were collected over a 2 ns time window at 40 ps resolution (50 gates) with the integration time of the camera set to 50 ms and the MCP voltage of 570 V. The measurements at the excitation wavelength for the complete set were acquired in 2.5 s. The fluorescence signals were recorded over a 4.6 ns time window at 40 ps resolution (115 gates) with the integration time of 800 ms and MCP voltage of 570 V. The emission measurements were acquired in 92 s.

### Fluorescence Lifetime Fitting Model

In this work, the lifetime distribution of the FRET-complex components was analyzed using a bi-exponential decay model wherein we estimated 4 unknown parameters – lifetimes and relative abundances of the FRETing and non-FRETing component of the donor molecules in the sample. For the temporal fluorescence decay function Γ_em(t),_ recorded in the absence of a diffusing medium on a system with instrument function response IRF(t), the biexponential decay model is given by:
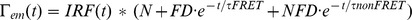



Here FD and NFD are corresponding to FRET donor fraction and non-FRET donor fraction, τ_FRET_ and τ_nonFRET_ are the lifetime values for the two species. Also, N is an arbitrary additive factor introduced to account for the noise in the fluorescence decay curve. The measured fluorescence decay (from 95% to 1% of peak intensity leading typically to ∼85 gates) is fit to this model and the above four parameters are estimated using a nonlinear constrained minimization method based on sequential quadratic programming, fmincon (MATLAB, MathWorks, USA).

The fitting process, for different A:D ratios, was carried out in two steps. First, the fluorescence decay of donor only (A:D = 0∶1) was fit with unbound lifetime estimates. The mean value of long lifetime (unquenched donor) was then selected as a fixed parameter for the second step and τ_FRET,_ FD and NFD were estimated. The lifetime of the NFD and FD were estimated to be 1.1±0.1 ns and 0.30±0.05 ns respectively. It should be noted that R-square for each fitting curve is around 0.99.

### Animal Experiments

Athymic nude female mice (6–12 week old) (BALBNU) from Taconic were used for live imaging experiments. Cells were plated onto 100 mm cell culture dishes and grown to between 1.7−1.0×10^6^ cells per dish. After the Tfn washout, cells were treated with AF700-Tfn and/or AF750-Tfn at molar ratios of 0∶1, 1∶2, 1∶1, or 2∶1 for 1 hour. Following uptake of NIR dye-conjugated Tfn, cells were washed in PBS and fixed with 4% formaldehyde for 15 minutes and suspended into 300 µL of matrigel substrate (BD, 356237). Cells are injected into the mammary fat pads of mice using a sterile 1 mL syringe and 27½ gauge needle (BD, 309623). Mice imaging was performed 3 hours post injection under vapor anesthesia (EZ-SA800 System, E-Z Anesthesia) using isofluorane and monitored using a physiological monitoring system (MouseOx Plus, STARR Life Sciences Corp.). Body temperature was maintained by an air warmer (Bair Hugger 50500, 3M Corporation) are integrated. All animal protocols were conducted with approval by the Institutional Animal Care and Use Committee at both Albany Medical College and Rensselaer Polytechnic Institute.

For tumor studies, mice were injected with 1×10^6^ live T47D cells re-suspended in 150 µL of growth media and 150 µL of matrigel substrate and allowed to grow for approximately 4–6 weeks. Following visible tumor growth, AF700-Tfn and/or AF750-Tfn at molar ratios of 0∶1 and 2∶1 were tail injected into mice with donor levels held constant at 40 µg/ml. After 1 hour post-injection, mice were anesthetized and imaged for the detection of FLIM FRET in live tumors.

## Results and Discussion

### Characterization of the Internalization of Tfn-Tfn-R Complexes into Cancer Cells using Intensity-based Confocal Microscopy FRET

The TfnR is expressed by many normal cells due to its critical role in the delivery of iron into cells. Specifically, TfnR is a homodimer that binds iron-loaded Tfn at the plasma membrane; subsequently, these Tfn-TfnR complexes are internalized into cells via clathrin mediated pits [43]. The release of Tfn from TfnR is achieved at the extracellular space upon return to the plasma membrane through the endocytic recycling pathway [44]. Due to their increased proliferation rates, which are iron-dependent, cancer cells express TfnR at significantly higher levels than normal cells [12], [45]. Therefore, Tfn has been widely used as a carrier for anti-cancer drugs as well as a probe for molecular imaging [14], [21]. Previously, the use of AF488-Tfn as a FRET donor that can effectively transfer energy to AF555-Tfn acceptor molecules upon internalization of Tfn-TfnR complexes into cells stably over-expressing human TfnR using intensity-based confocal microscopy has been reported in detail [23]–[27], [41]. Here, for the first time, the Tfn system has been used to determine FRET between donor- and acceptor-Tfn bound to endogenous TfnR homodimers to quantify Tfn binding and internalization into cancer cells.

The TfnR expression levels have been characterized in T47D breast cancer cells and compared to similarly derived HMECs. TfnR protein levels (Figure S1 in File S1), are higher expressed in T47D cancer cells in comparison to the HMECs. To test whether a higher TfnR expression level correlated with increased Tfn internalization in cancer cells, a functional analysis of TfnR expression was performed using a Tfn uptake fluorescence imaging-based assay. Using a time course experiment, we concluded that T47D cells show a significantly increased total amount of Tfn internalization compared to normal HMECs at the indicated time points (Figure S2 in File S1). Furthermore, Tfn is internalized at higher levels at early time points in T47D cells vs. HMECs.

To quantify bound and internalized Tfn, we utilized an intensity-based quantitative confocal microscopy FRET *in vitro* assay based on the binding of acceptor- and donor-labeled Tfn to the homodimeric TfnR at the plasma membrane. This quantitative FRET assay determines acceptor, quenched and unquenched donor fluorescence intensities, as well as the E% between the excited donor fluorophore and acceptor fluorophore. E% is calculated as one minus the ratio of quenched donor to unquenched donor signal in percent form [26], [27], [36]. E% measurements against A:D ratios and acceptor intensity levels have been used to determine whether the FRET signal is due to random interactions or specific clustering [38], [39], [42], [46]. Previously, we have shown that Tfn-TfnR complexes showed E% levels that were positively dependent on A:D ratios and independent of acceptor levels, confirming a clustered distribution of Tfn-TfnR complexes (including intramolecular interactions between Tfn molecules bound to homodimeric TfnR and putative higher-order clusters of internalized Tfn-TfnR complexes) upon internalization of donor (AF488) and acceptor- (AF555) labeled Tfn into epithelial MDCK (Madin-Darby canine kidney) cells using both intensity-based confocal microscopy and FLIM two-photon microscopy [23].

**Figure 1:**
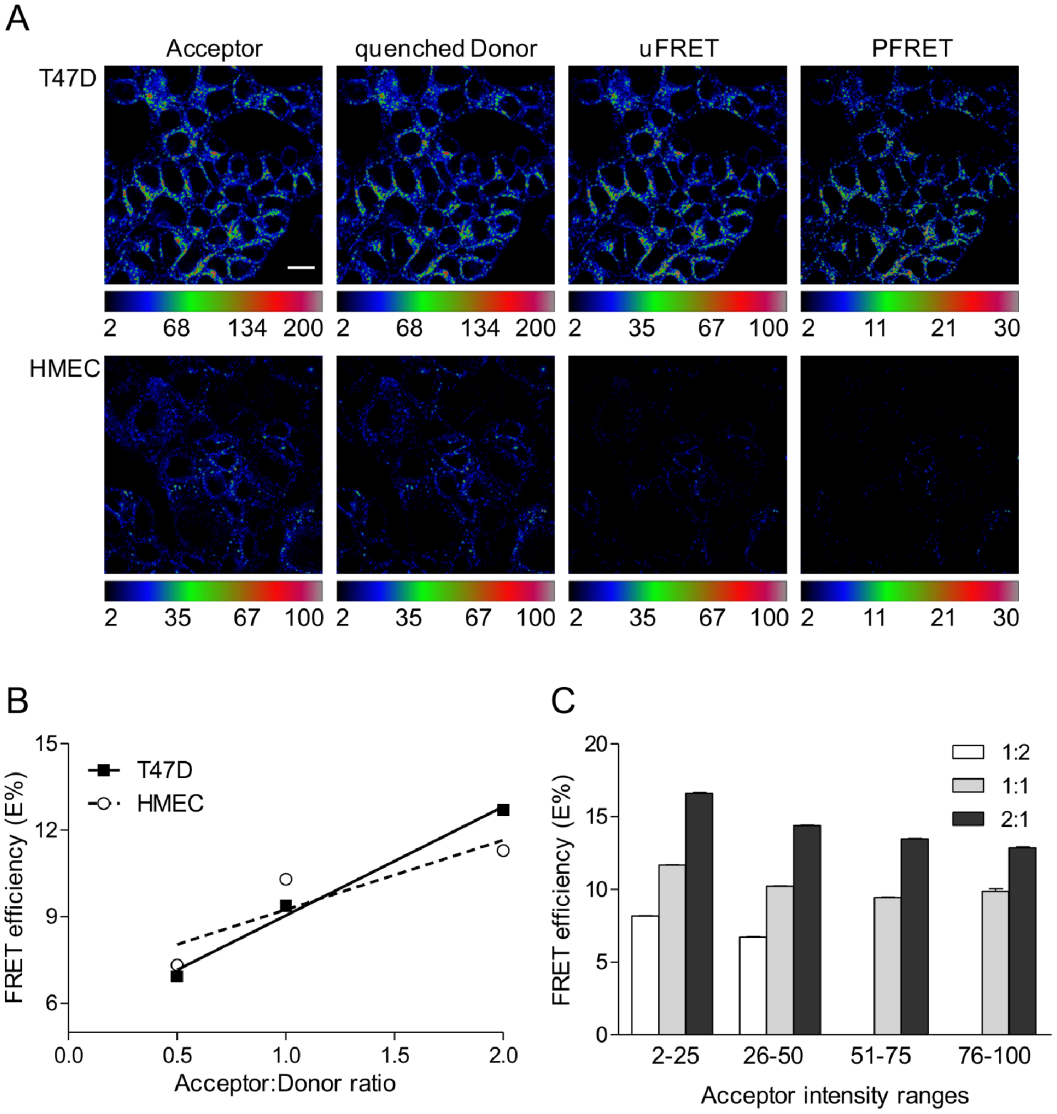
Characterization of Tfn-Tfn-R complexes in normal vs. cancer cells using intensity-based confocal microscopy FRET. Tfn-TfnR FRET assay shows similar behavior in T47D breast cancer cells and HMECs. (A) T47D breast cancer cells have significantly more uptake of AF488-Tfn and AF555-Tfn compared to HMECs, as shown in both the acceptor (AF555) and quenched donor (AF488) channels. Pseudocolor images of acceptor, quenched donor, uncorrected FRET (uFRET) and corrected FRET (PFRET) show the typical endocytic pattern of irregular and punctate structures. PFRET images confirm the existence of energy transfer between endogenous Tfn-AF488 (donor) and Tfn-AF555 (acceptor) in intracellular punctate endocytic structures. Scale bar = 20 µm. (B) E% is calculated and plotted against internalized A:D ratios of 1∶2, 1∶1, and 2∶1 in T47D and HMECs. Despite lower FRET signal in HMECs, a trend for higher E% relative to increasing A:D ratios is clearly seen in both HMECs and T47D cells. (C) E% is plotted against binned acceptor intensity levels. As A:D ratios increase, E% increases. Statistical analysis of this relationship is shown in Table S1 in File S1. However, within each A:D ratio, E% is independent of acceptor intensity levels in T47D cells (Table S2 in File S1). Together with the trend shown in (B), these patterns support a pattern consistent with a clustered organization of TfnR-Tfn complexes rather than random interactions. Error bars indicate 95% confidence interval.

The quantitative intensity-based FRET assay was performed in cancer vs. normal cells using confocal microscopy to determine whether Tfn-TfnR complexes show a clustered behavior upon internalization of Tfn into cells. Iron-bound Tfn molecules were exogenously conjugated to AF488 or AF555 dyes as donor and acceptor molecules, respectively, and then were internalized at different A:D ratios into T47D cells vs. HMECs for 60 minutes at 37°C. This approach allows for the control of the internalized A:D ratios, which have been shown to correlate with estimated A:D ratios calculated from acceptor and donor gray-scale intensity levels generated by the intensity-based FRET assay (see Methods section) [24]. After fixation, cells were imaged using a FRET confocal microscopy methodology, as described previously [23], [35]. Here, A:D ratios were experimentally set at 1∶2, 1∶1, and 2∶1. These ratios were validated post-experimentally and shown to correlate to the A:D values calculated from pixel intensity measurements using custom generated analytical FRET correction software (PFRET software) as described in detail [23], [35]. Representative examples of confocal FRET pseudo-color images using an A:D molar ratio of 2∶1 AF555-labeled Tfn to AF488-labeled Tfn molecules are shown in Fig. 1A. HMEC images are shown using a narrower intensity pseudo-color scale for illustrative purposes but are quantitatively analyzed using identical conditions to those applied to T47D cells. In the acceptor and quenched donor images, both T47D cells and HMECs show Tfn uptake, although the T47D cells show significantly higher levels of Tfn internalization vs. control HMECs (Fig. 1A). A typical endocytic pattern is shown in both the cancer and normal cells. Uncorrected FRET (uFRET) and subsequent corrected FRET, or precision FRET (PFRET) images were obtained after appropriate background subtraction and spectral bleed-through corrections using the PFRET software [23], [26], [27], [36]. As expected, given the Tfn uptake differences, uFRET and PFRET images also show increased levels in T47D cells vs. HMECs (Fig. 1A, right column). Nevertheless, energy transfer between donor and acceptor labeled Tfns bound to TfnR are seen in both normal and cancer cells (Fig. 1A).

To perform the quantitative intensity-based FRET confocal microscopy assay, a wide variety of 10×10 pixel regions of interest (ROIs) were selected and average values for E%, donor and acceptor intensities were calculated as described previously [23], [26], [27], [36]. E% levels split by A:D internalized ratios are similar at each A:D ratio between T47D cells and HMECs as shown in Fig. 1B. In both cell types, the E% values increase similarly as the ratio of A:D ratios increases (T47D slope = 3.770±0.3906, HMEC slope = 2.410±1.226, p = 0.4014, Table S1 in File S1). As shown in Fig. 1C, E% does not show a positive dependency on acceptor levels, remaining relatively constant in T47D cells at fixed A:D levels, demonstrating a clear independence of E% versus acceptor behavior. Mathematical models have shown that during random interactions, E% is positively related to acceptor intensities at fixed A:D ratios. Conversely, in clustered interactions, E% is positively related to increasing A:D ratios [24], [38], [39]. Based on our findings, we report that TfnRs in T47D cancer cells and normal HMECs are organized into a clustered pattern, as expected due to the homodimeric nature of TfnR and clustered behavior of receptor-ligand complexes throughout endocytic trafficking pathways [12]. Furthermore, despite a significantly higher expression level of TfnR in T47D cells, these interactions remain organized into clusters without significant increase in FRET levels occurring between TfnR clusters. However, a clear difference between cancer and normal cells can be detected when comparing the FRET level of Tfn-TfnR complexes at higher vs. lower levels of internalized acceptor AF555-Tfn molecules (Fig. S3 in File S1). T47D cancer cells show high FRET levels at a wide range of acceptor AF555-Tfn intensity levels. However, due to the relatively lower level of Tfn uptake, normal HMECs only show detectable FRET signals at low ranges of acceptor AF555-Tfn intensities (Fig. 1C vs. Fig. S3 in File S1). These results demonstrate that differences in TfnR expression levels between cancer vs. normal cells is reflected in the detection of FRET at higher intensity levels of acceptor labeled Tfn. They also establish that intensity-based TfnR-Tfn FRET assay can be used to quantitatively distinguish cells expressing high levels of TfnR found in cancer cells from non cancerous cells that show lower levels of TfnR expression. Therefore, we are able to discriminate cancer cells from normal cells by thresholding FRET at higher acceptor intensity levels.

### Fluorescence Lifetime Provides a Robust Measurement of FRET using NIR Fluorophores *in vitro*

We have utilized NIR FLIM FRET to quantify cellular Tfn internalization in cancer cells using a wide-field time resolved imaging platform. Because our main goal is to detect Tfn uptake by cancer cells using FRET measurements *in vivo* through a small animal model in a non-invasive manner, we developed a new approach to perform FRET assay *in vivo* through tissues, up to a couple of centimeters. First, we conjugated iron bound Tfn to NIR fluorophores based on their ability to deeply penetrate living tissues compared to visible-wavelength fluorophores while maintaining high signal-to-noise ratios. Second, we conjugated Tfn molecules to AF700 (donor) or AF750 (acceptor) NIR fluorophores that possess a strong R_0_ value and sufficiently prolonged donor excitation decay times to achieve accurate fluorescence lifetime FRET measurements, i.e. AF700/AF750 FRET pair with a R_0_ = 7.76 nm and τ = 1100 ps. Third, the ability to image and analyze data *in vitro* from 96-well plates at fast acquisition speed (<2 minutes) was achieved by using active wide-field illumination in a time-domain fluorescence lifetime imaging system, which is based on gate intensified CCD detection (see Methods section).

**Figure 2:**
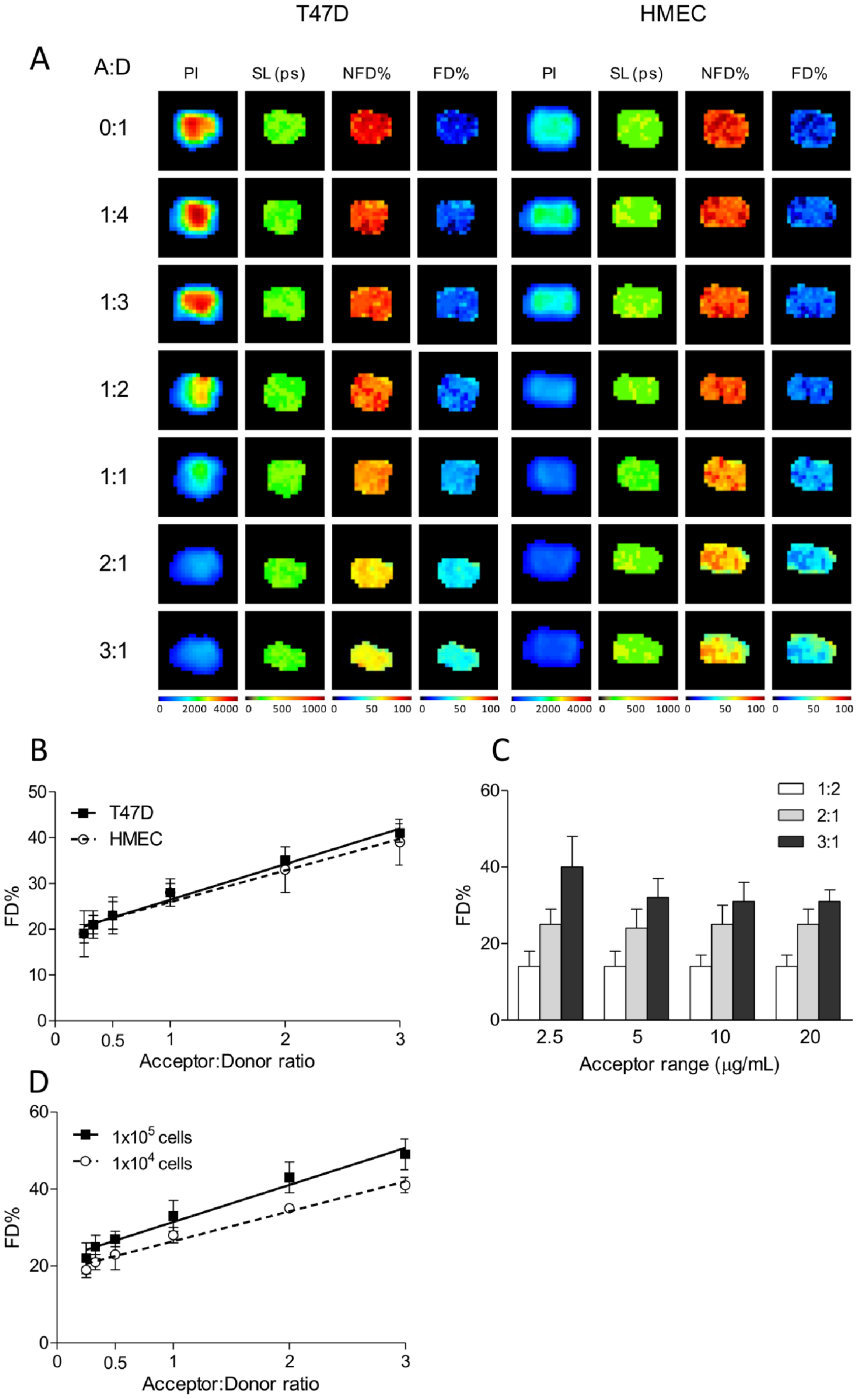
NIR fluorescence lifetime FRET of TfnR-Tfn complexes in normal vs. cancer cells using time-domain, wide-field macroscopic imaging *in vitro.* Fluorescence lifetime analysis of TfnR-Tfn complexes using NIR FRET pair AF700–AF750 and a time-domain, wide-field macroscopic imaging system. (A) T47D (left panels) and HMECs (right panels) were internalized with AF700-Tfn (donor) or AF750-Tfn (acceptor) in increasing A:D ratios of 0∶1, 1∶4, 1∶3, 1∶2, 1∶1, 2∶1, and 3∶1 in a 96-well format. The first column on the left indicates the pixel intensity (PI) of donor fluorophores (AF700-Tfn) and the subsequent decrease in intensity as the ratio of acceptor molecules (AF750-Tfn) increase due to quenching. Short component lifetimes (SL) measured in picoseconds (ps) are shown in the second column, indicating a high sensitivity and uniform detection despite the heterogeneity of donor intensities in the sample (Table S3 in File S1). The columns on the left indicate the relative abundance of NFD populations and FD populations. Each image shows 20×20 pixels with 0.5 mm/pixel size. (B) Quantification of donors participating in FRET events are shown as %FD and show a positive relationship relative to increasing proportion of acceptor molecules in both T47D and HMECs (Table S4 in File S1). (C) The %FD in relation to acceptor levels show an independent relationship as seen in FRET results using visible FRET detected by confocal microscopy (Table S5 in File S1). (D) Sensitivity of FD% is consistent across 1×10^5^ to 1×10^4^ cells (Table S6 in File S1). PI = pixel intensity, SL = short lifetime, NFD% = non-FRET Donor%, FD% = FRET Donor%. Error bars indicate standard deviation.

As this is the first description of NIR FLIM FRET to detect Tfn uptake by cancer cells, we validated NIR FLIM FRET results against data generated by intensity FRET confocal microscopy (Fig. 1). In Fig. 2A, T47D and HMEC grown in 96-wells were treated with Tfn conjugated to NIR flurophores (AF700/AF750 as donor and acceptor NIR fluorophores respectively) for 60 minutes at increasing A:D molar ratios starting from donor only and progressing from 1∶4, 1∶3, 1∶2, 1∶1, 2∶1, to 3∶1. Using the wide-field illumination macroscope imaging platform, fluorescent lifetime analysis and mathematical modeling, allows the estimation of the relative contributions of the non-FRET donor (NFD), i.e. unquenched donor population and FRET donor (FD), i.e. quenched donor population. As the relative ratio of acceptor molecules (AF750-Tfn) increases, the subsequent contribution of the short lifetime component of the donor molecule (AF700-Tfn) during its excited state should increase.

**Figure 3:**
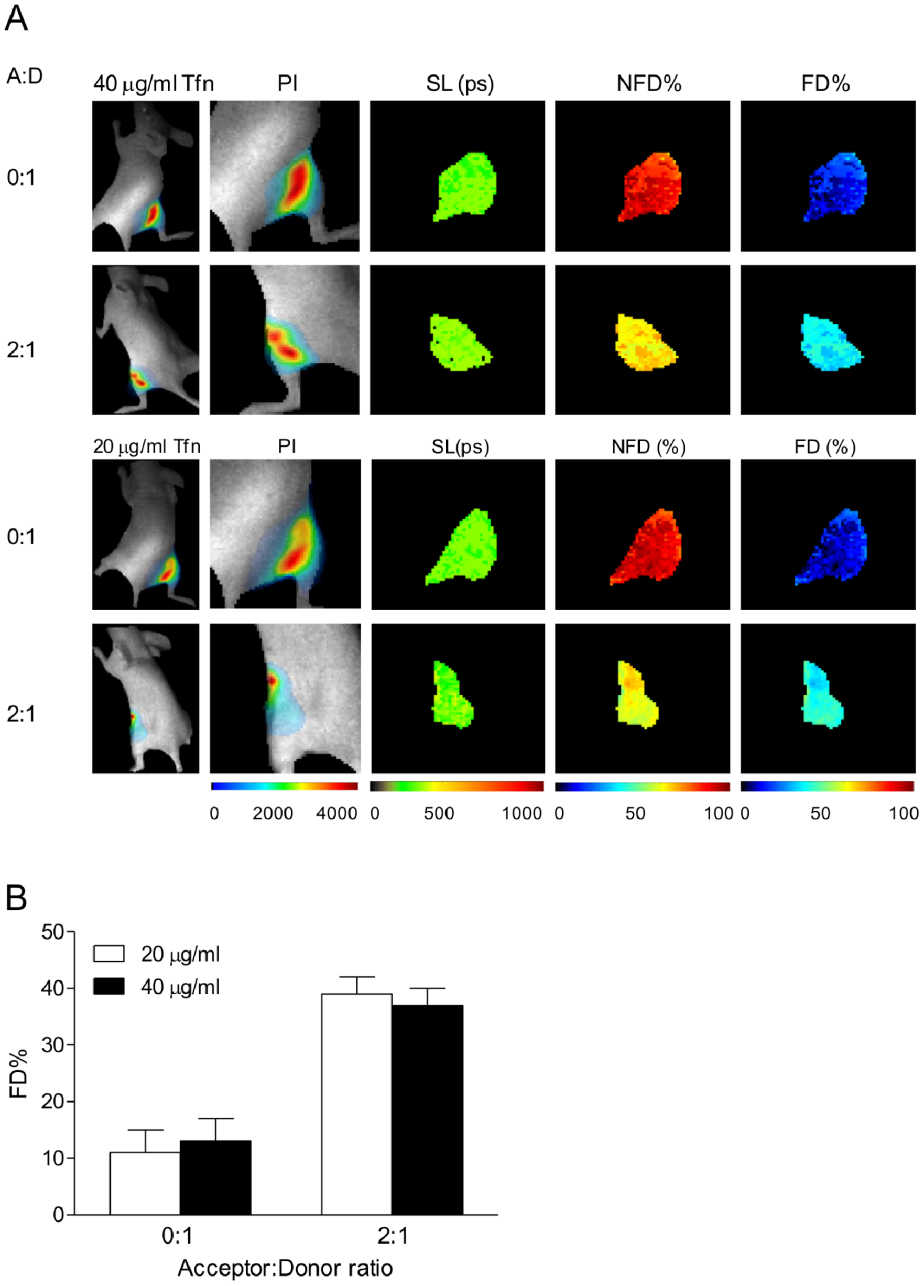
NIR fluorescence lifetime FRET of TfnR-Tfn complexes in normal vs. cancer cells using wide-field macroscopic imaging *in vivo.* Fluorescence lifetime FRET is detected between AF700-Tfn (donor) and AF750-Tfn (acceptor) in T47D cells within a live mouse. (A) Mice injected with ∼1.5×10^6^ T47D cells internalized with various ratios of NIR dyes conjugated to Tfn are shown. In the top two rows, donor AF700-Tfn is held constant at 40 µg/ml and imaged. Pixel intensity (PI) is shown on the first column on the left of the donor fluorophore. Short component lifetime (SL) of the donor dye (Table S7 in File S1) is uniform among a varied intensity of donor dye. The next two columns on the right show the population of non-FRET donor (NFD) and FRET-donor (FD), respectively, showing an increase upon introduction of the AF750-Tfn (acceptor). Similar results are shown with Tfn-AF700 (donor) at 20 µg/ml. Each image shows 60×60 pixels with 0.5 mm/pixel size. (B) Results of the FD% show similar results between the two amounts, and also showing similar increases in FD in relation to the addition of AF750-Tfn acceptor (Tables S8–S10 in File S1). Error bars indicate standard deviation. PI = pixel intensity, SL = short lifetime, NFD% = non-FRET Donor%, FD% = FRET Donor%.

**Figure 4:**
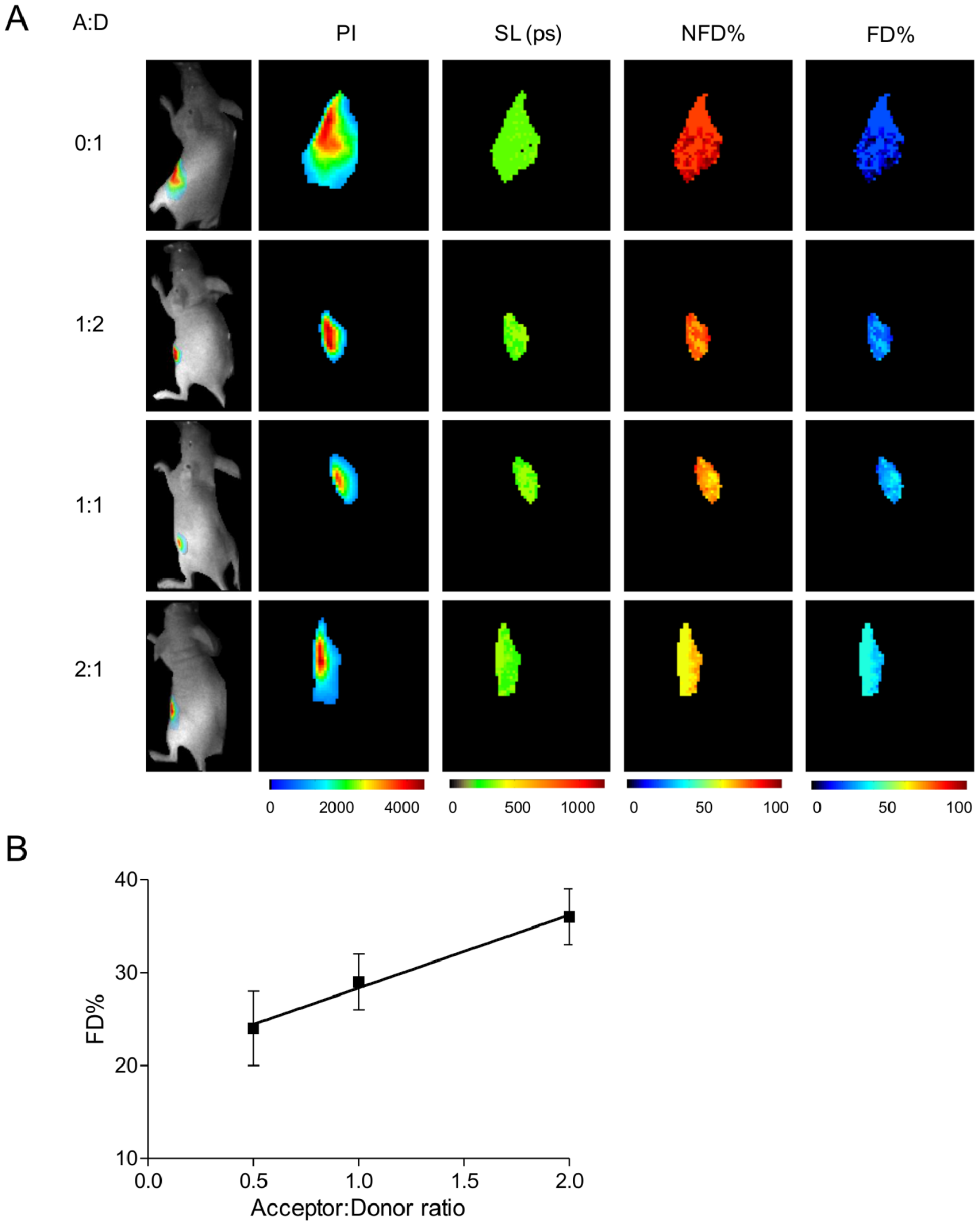
Accuracy of fluorescence lifetime FRET using NIR fluorophores *in vivo.* Decreased fluorescence lifetime due to FRET is detectable through living mice. (A) Mice are injected with matrigel resuspended ∼1×10^6^ cells pre-internalized with Tfn conjugated with NIR dyes at A:D ratios of donor only, 1∶2, 1∶1, and 2∶1 and imaged. Images show donor intensities on the left column followed by measurements of the short lifetime component (Table S11 in File S1), which also shows uniformity. The NFD and FD species are shown on the following columns to the right. Each image shows 60×60 pixels with 0.5 mm/pixel size. (B) Quantification of the FD% shows a robust linear increase in proportion to an increasing amount of AF750-Tfn (acceptor). These results indicate that the macroscopic imaging system employed is capable of determining FRET-mediated lifetime changes of donor fluorophores in cells through living animal tissues. Error bars indicate standard deviation.

The estimation of the short lifetime component and associated estimation of NFD vs. FD populations are shown in Fig. 2A. These results demonstrate that the imaging methodology is robust and sensitive to NIR lifetime donor reduction upon FRET, with the short lifetime estimation being homogenous despite the high dynamical range in the pixel intensity of the samples. The homogenous behavior of the short lifetime component (Table S3 in File S1) suggests a narrow range of distances within the TfnR-Tfn complex binding and internalization processes, which results in a tight range of E% levels both as seen using intensity-based confocal microscopy (Fig. 1), two-photon fluorescence lifetime microscopy (FLIM), and macroscopy FLIM methods (Figs. 2, 3, 4) [23], [26]. Moreover, the larger pixel size (0.5 mm/pixel) in macroscopic lifetime imaging results in the “averaging” of lifetime values in an ensemble-like fluorescence lifetime analysis. As seen in Figs. 2B and 2D, increasing A:D ratios results in a linear increase in the FD% in both T47D and HEMCs; no statistical differences in the positive slopes for both cell types are detected (T47D = 7.767±0.5321 and HMEC = 6.896±0.5845, p = 0.303, Table S4 in File S1). These results are consistent in terms of relative differences with the results shown in Fig. 1B. In Fig. 2C, the FD% shows a stepwise increase as A:D ratios increase in T47D cells but within each ratio, the FD% remains constant as acceptor concentrations increase, and is comparable to the analogous experiment in Fig. 1C using intensity-based FRET(Table S5 in File S1). This provides further evidence that despite an overall decrease in uptake of Tfn in HMECs compared to T47D cells, the pattern of FD% vs. A:D ratios and the acceptor concentration ranges indicate a clustering pattern of organization. Taken together, these results are entirely consistent with the results shown in Fig. 1.

To test detection sensitivity of the wide-field illumination imaging platform, we compared cell densities using 1×10^5^ and 1×10^4^ cells plated onto 96-well plates and incubated with identical amount and ratios of acceptor and donor NIR Tfn in Fig. 2D. The increase in FD% against the A:D ratios provided positive slopes for both plating densities (1×10^5^ cells = 9.631±0.7624 and 1×10^4^ = 7.767±0.5321, p = 0.08, Table S6 in File S1) that are not statistically distinguishable. From these results, we conclude that the FRET results obtained by measuring the decrease of NIR donor lifetimes using wide-field illumination platform are robust, sensitive, and quantitatively consistent with FRET data obtained using visible fluorophores under intensity-based confocal microscopy. These results strongly validate the potential of NIR FRET FLIM for quantitative assays and lay the foundation to investigate if cancer cells could be detected through a live small animal using FRET signals.

### Fluorescence Lifetime Provides a Sensitive Measurement of FRET using NIR Fluorophores *in vivo*

Using a live small animal model, our initial aim was to first achieve detection of NIR FLIM FRET through living tissues in nude mice, and secondly, to determine sensitivity of the NIR FRET FLIM approach using low doses of NIR labeled Tfn. T47D cancer cells were pre-loaded with Tfn using either donor only or an A:D ratio of 2∶1 to determine if a reduction of lifetime could be detected through living tissue. After internalization, T47D cells were re-suspended into matrigel and then injected into the ventral areas of the mice. In Fig. 3A, the top row shows T47D cancer cells in a mouse revealing the distribution of the signal intensities on the left column followed by the detection of the short component lifetime. As seen previously, the detection of the short lifetime components (measured in ps, Table S7 in File S1) is highly sensitive, and remains homogenous despite heterogeneity of pixel intensities (due to heterogeneous optical properties and tissue thicknesses). In the absence of acceptor, the relative NFD% remains very high. However, upon addition of acceptor using an A:D ratio of 2∶1 respectively, detection of FD increased in proportion to the NFD, indicating that FRET events occurring between Tfn molecules are detected in real time using NIR FLIM *in vivo*. Next we wanted to determine if FRET, through a reduction in donor lifetime, could be detected using half the amount of Tfn (20 µg/ml) (Tables S8–S10 in File S1). FRET is still detectable compared to higher levels of internalized Tfn without any loss of detection accuracy as shown in the quantified data (bottom row) in Fig. 3B. These results verify that NIR FLIM FRET imaging is capable of sensitive detection of Tfn-mediated FRET signal through living animal tissues.

### Fluorescence Lifetime Provides an Accurate Measurement of FRET using NIR Fluorophores *in vivo*

After establishing that we can detect FRET using NIR fluorophores in mice, we sought to confirm the quantitative accuracy of this method *in vivo*. Accordingly, mice were injected with T47D cells pre-internalized with varying A:D ratios of NIR-labeled Tfn, ranging from donor alone, 1∶2, 1∶1, to 2∶1 ratios, respectively. These ratios were used to determine if linearity of FD fractions could also be detected through living tissues. T47D breast cancer cells were orthotopically injected in a matrigel matrix as described in the Methods section. Following solidification of the matrix *in vivo*, mice were imaged under anesthesia for detection of donor fluorophore lifetime reduction. As shown in Fig. 4A, we are able to detect an increase in the relative proportion of FD in the presence of increasing amounts of acceptor. Quantitative analysis of the data (Fig. 4B) shows a linear increase in FD fractions in proportion to increased acceptor levels, (slope = 7.857±0.7423, R^2^ = 0.9912), with significant differences between A:D ratios of 1∶2 and 2.1 with a p-value <0.0001 using t-test (see Table S12 in File S1). These *in vivo* results are consistent with the *in vitro* data and demonstrate that our imaging platform is sensitive and quantitatively matches *in vitro* results generated by confocal microscopy. This new approach to image, detect and quantify nanometer scale information about the intracellular organization of membrane-bound receptor-ligand complexes represents a significant advance towards the non-invasive quantitation of the internalization of Tfn-based targeted delivery systems *in vivo*.

### Fluorescence Lifetime Reduction due to FRET is Detectable within Live Tumors *in vivo*

**Figure 5:**
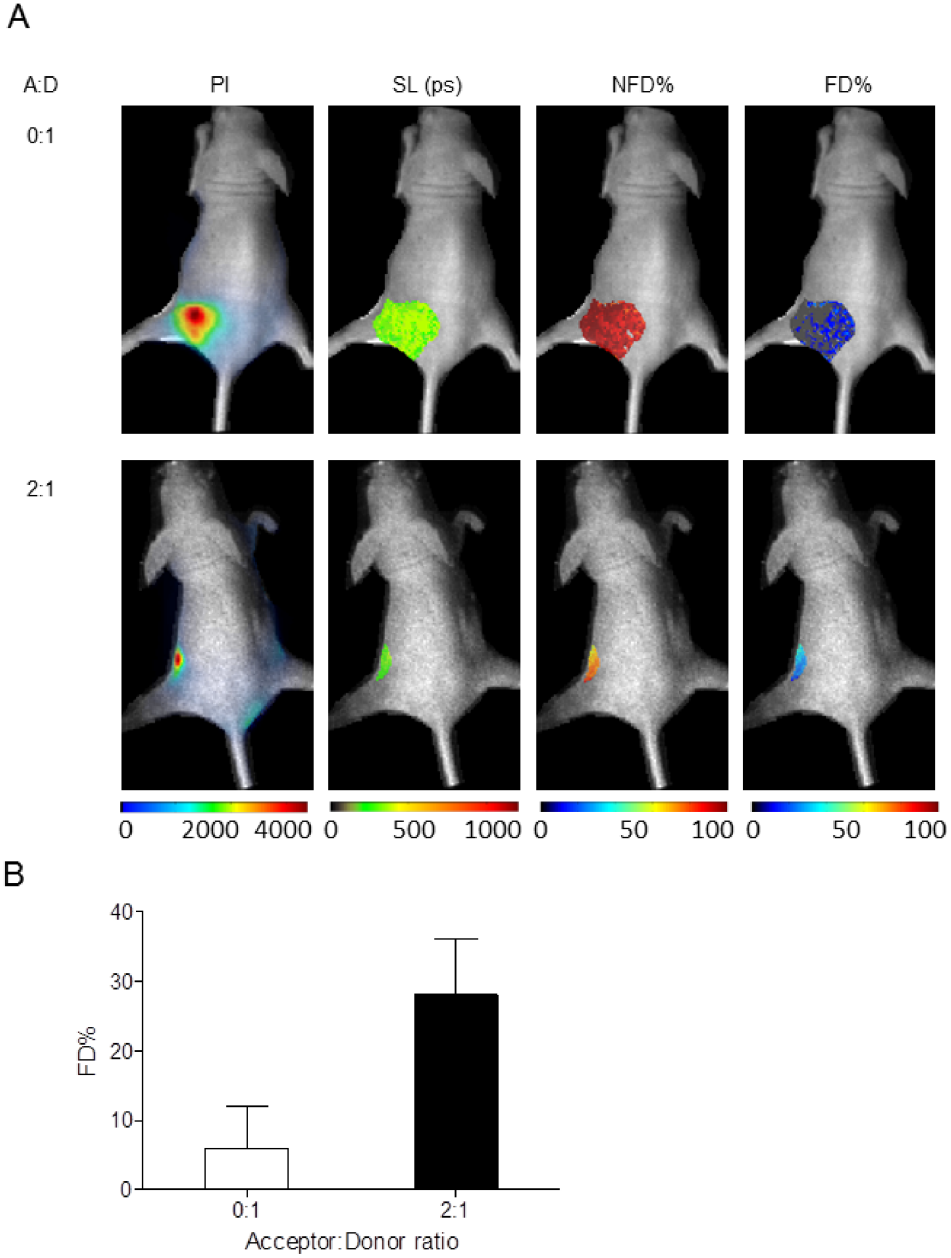
NIR fluorescence lifetime FRET of TfnR-Tfn complexes in tumor xenografts using wide-field macroscopic imaging *in vivo.* Detection of decreased fluorescence lifetime due to FRET is achieved within mouse tumor model. (A) Mice implanted with live T47D cells developed tumors after 4–6 weeks of growth. For FRET detection, mice were injected with Tfn conjugated with NIR dyes using A:D ratios of 0∶1 (upper row) or 2∶1 (lower row) and subjected to fluorescence lifetime FRET imaging 1 h post-injection. Panels from left to right show pixel intensity (PI), the short lifetime (SL) estimation and distribution, non-FRET donor % (NFD%) and FRET donor % (FD%). (B) Quantification of FD% indicates a higher proportion of FD% in the presence of acceptor NIR-Tfn, thus demonstrating the capability to detect FRET between AF700-Tfn and AF75-Tfn bound to homodimeric TfnR in tumors within a live mouse model.

To further establish the feasibility of our approach, live T47D breast cancer cells were implanted into nude mice. After 4–6 weeks of tumor growth, mice were given AF700-Tn and/or AF750-Tfn at ratios of 0∶1 and 2∶1 through tail vein injections and subjected to fluorescence lifetime FRET. As seen in Figure 5, an increase in %FD in mice is observed in mice given AF700-Tn and AF750-Tfn at an A:D ratio of 2∶1 compared to AF700-Tfn donor only treated mice at one hour post-injection, demonstrating a lifetime reduction due to FRET in live tumors *in vivo*.

FLIM FRET-based imaging technology does not allow absolute determination of the amount of NIR-labeled Tfn that is found in receptor-bound or unbound state. Rather, it describes the proportion of donor-labeled Tfn in the FRET-ing state (FD%) vs. that in the non-FRET-ing (NFD%) state. Whereas FD% includes Tfn-Tfn-R complexes at the plasma membrane and in intracellular endocytic membranes, NFD% includes extracellular, soluble and unbound donor NIR-Tfn. Thus, FD% indicates the fraction of intracellular and receptor-bound Tfn of total Tfn injected into the animal or added into cells. As indicated in Figure 5, upon tail vein injection of AF700- and AF750-Tfn at a A:D ratio 2∶1, FD% measurements suggest that ∼30% of total Tfn injected into mice is bound and internalized into tumor at 1 h post injection. These results as benchmarked against the Figure 3B and 4B data obtained with T47D cells pre-internalized with Tfn and immobilized in matrigel plugs, indicate the strength and reliability of fluorescence lifetime FRET to quantitate the relative amount of Tfn bound and internalized into cancer cells and tumors. As such, our technology is unique as it detects and quantifies *in vivo* non-invasively the relative proportion of receptor-bound and internalized Tfn of total added or injected into cancer cells or tumor xenografts. These results hold tremendous promise towards future applications of this technique in the optimization of targeted drug delivery systems by determining the intracellular amounts of Tfn-conjugated drugs labeled with acceptor and donor dyes, and thus establishing Tfn cellular residency parameters to monitor anti-cancer efficacy of Tfn-drug conjugates.

These results are the first to demonstrate quantitative *in vivo* FRET analysis of internalized Tfn in cancer cells and tumors in live animals. Our intensity-based *in vitro* FRET data is consistent with data acquired using wide-field illumination NIR fluorescence lifetime FRET *in vitro* and *in vivo*, establishing that we can exploit the homodimeric nature of the TfnR to monitor Tfn internalization. Hence, our approach allows for the discrimination between non-bound versus bound/internalized Tfn and provides a new method to accurately determine whether Tfn-conjugated drugs are internalized into cancer cells. Furthermore, fluorescence lifetime FRET can visualize and quantitate intracellular receptor-bound Tfn, which includes receptor bound-Tfn at the plasma membrane as well as during clathrin-mediated internalization and subsequent endocytic recycling pathway back to the plasma membrane. Since Tfn bound to its receptor can continue to FRET during the endocytic recycling of the Tfn-receptor complexes, our FRET based method should be able to provide a measure of the level of intracellular accumulation of Tfn-drug conjugates as it is widely recognized that internalization and endocytic recycling of Tfn-drug conjugates can affect the development of effective therapies [13]. We expect NIR fluorescence lifetime FRET to allow for the rapid screening of Tfn-drug candidates that show higher levels of intracellular accumulation in tumor cells, thus providing a rapid non-invasive screen of the more likely effective Tfn-drug candidates *in vivo*. This would be a crucial tool to further our ability to optimize targeted drug delivery strategies into regions that are prone to the EPR effect, such as tumors [3]. Given the ability to detect cancer cells with high sensitivity and to distinguish T47D cells from HMECs using both the intensity-base FRET confocal microscopy and the macroscope fluorescent lifetime imaging system, we propose that this system may provide also a diagnostic tool for early detection and characterization of neoplasm. Moreover, by applying a macroscopic, wide-field illumination platform, we were able to quantify FRET in live animals in a manner that is quantitatively consistent with established methods that are routinely used in FRET microscopy. Thus, our NIR fluorescence lifetime FRET approach offers the possibility to employ well-characterized quantitative metrics used in FRET microscopy, but with the additional benefit of a seamless technological platform to perform quantitative *in vitro* and *in vivo* assays. Moreover, these results combined with previous work [32], [47], [48] lay the foundation for establishing quantitative 3D tomographic imaging using FRET. Having such capabilities at our disposal would be a tremendous tool to non-invasively quantify tumor sizes and/or regions of active uptake of the labeled ligand with accuracy. Since our approach may be extended to quantitatively image the interaction of numerous receptor-ligand complexes *in vivo*, it could provide a critical tool to discern protein-protein interactions in the native *in vivo* environment. In doing so, it would confer a tremendous increase in understanding the true physiological mechanisms underlying diseases such as cancer and/or to optimize drug delivery and assess therapeutic efficacy *in vivo*. In summary, we present data that supports the quantitative accuracy and sensitivity of NIR fluorescence lifetime FRET to be used non-invasively through living tissues in a small animal model.

## Supporting Information

File S1 (PDF)
