# Supplementary material for: Non-Invasive In Vivo Imaging of Near Infrared-labeled Transferrin in Breast Cancer Cells and Tumors Using Fluorescence Lifetime FRET
Source: PLoS One. 2013 Nov 21;8(11):e80269. doi: 10.1371/journal.pone.0080269 (PMC3836976; doi:10.1371/journal.pone.0080269)
Supplement: File S1 — (PDF) [file pone.0080269.s001.pdf]

Supplementary Figures and Tables

Abbreviations: Tfn: transferrin, TfnR: transferrin receptor, A: acceptor, D: donor, AF: AlexaFluor, FRET: Förster Resonance Energy Transfer, E%: energy transfer %, EPR, , HMEC: human mammary epithelial cell, FD: FRET donor SD: standard deviation

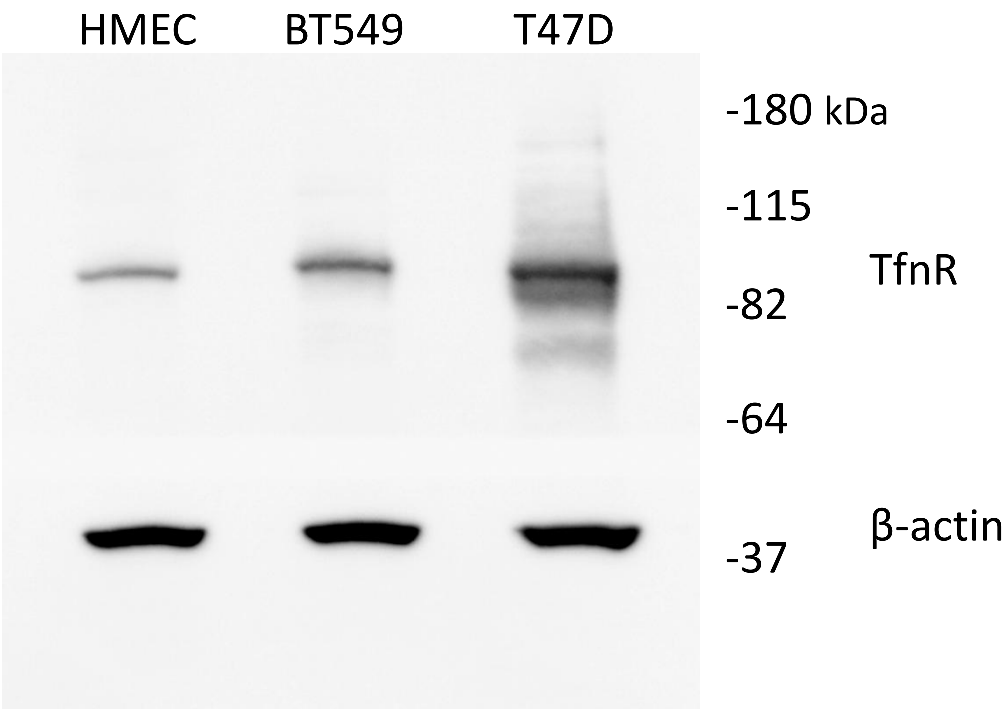

Supplementary Figure S1. Western Blot of TfnR in HMECs, BT-549, and T47D cells. Blots indicate higher expression of TfnR in human breast cancer cell lines BT549 and T47D compared to normal HMECs

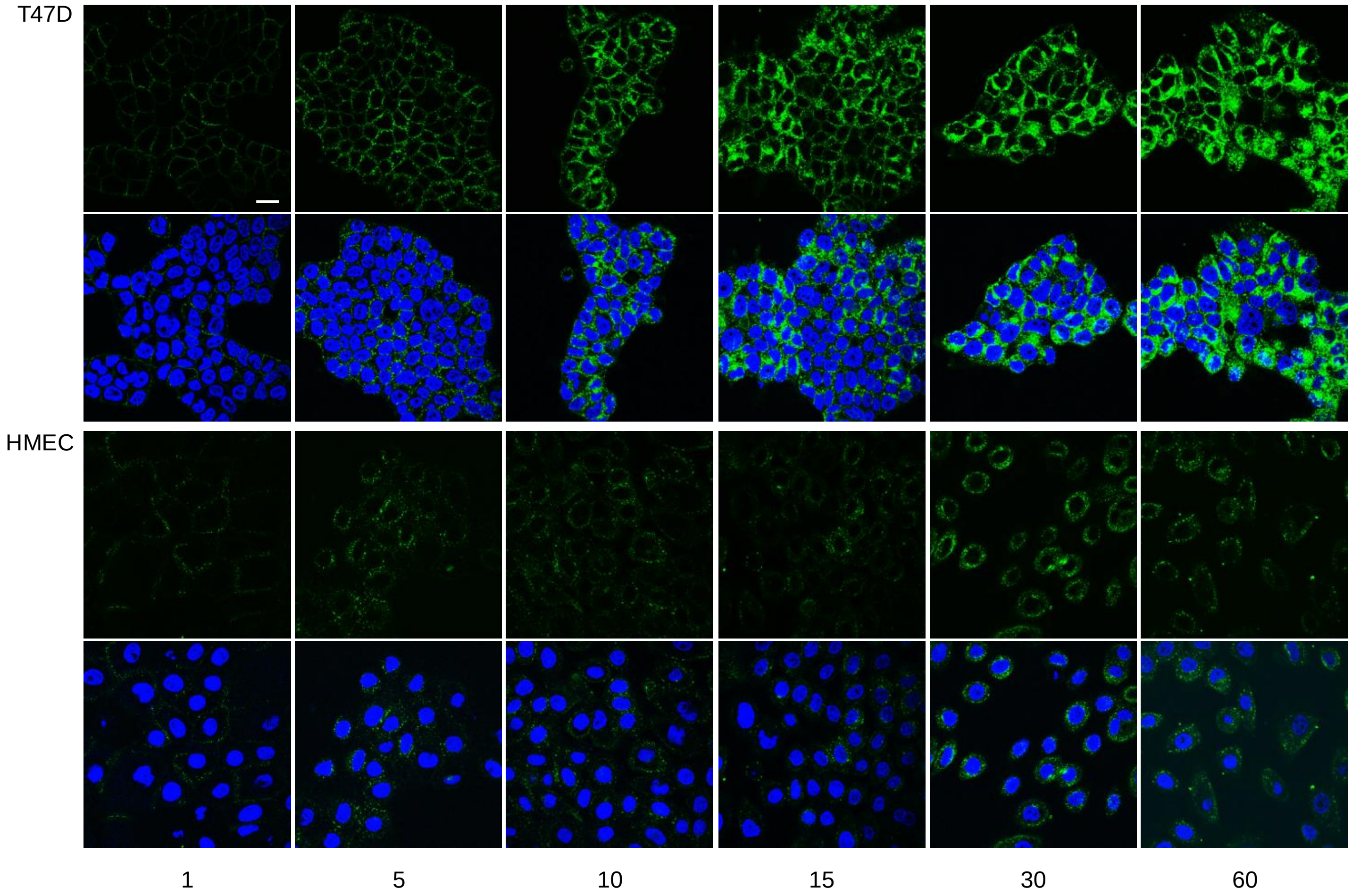

Supplementary Figure S2. HMEC and T47D cells are incubated with Tfn conjugated to AF488 (green) for the times as indicated (minutes) and imaged under confocal microscopy with DAPI staining (blue). Scale bar = 20 μm. Tfn-AF488 internalization into HMEC and T47D cells is clearly detected between 1 and 10 min, showing plasma membrane localization at 1-5 min and intracellular peri-nuclear staining at 10-60 min.

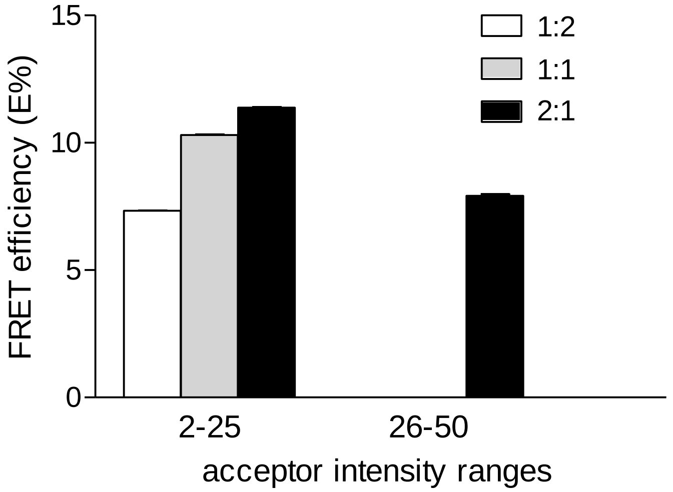

Supplementary Figure S3. HMEC cells internalized with A:D ratios of 1:2, 1:1, and 2:1 and plotted with binned acceptor intensity per pixel and E% values. Error bars indicate 95% confidence intervals. Absence of higher acceptor intensity ranges reflect decreased uptake of Tfn. A:D ratios of 1:2 and 1:1 in the 6-50 range is likely due to higher ratio of acceptor quenching donor. Similar increase in E% as A:D ratios increase (at acceptor range of 2-25) is observed compared to results found in Fig. 1B (main text).

Supplementary Table S1 (for Figure 1B)

Cell Type	T47D	HMEC
Slope	3.770 ± 0.3906	2.410 ± 1.226
95% CI for Slope	-1.193 to 8.732	-13.17 to 17.99
r^2	0.9894	0.7943
p-value	0.401 (n.s.)	
analysis method	ANCOVA	

Statistical analysis of slopes of FRET efficiency (E%) vs. A:D ratios between T47D and HMECs. No statistical significance is detected between T47D and HMEC slopes using Ancova analysis, suggesting that TfnR-Tfn complexes show similar FRET behavior in HMEC vs. T47D cells. n.s. = not statistically significant (p>0.05)

Supplementary Table S2 (for Figure 1C)

Acceptor Range (pixel intensity)	0-25			26-50			51-75			76-100		
A:D ratios	1:2	1:1	2:1	1:2	1:1	2:1	1:2	1:1	2:1	1:2	1:1	2:1
Average (E%)	8.183	11.682	16.613	6.725	10.230	14.400	ND	9.434	13.476	ND	9.871	12.857
95% CI	0.140	0.149	0.391	0.376	0.138	0.168	ND	0.376	0.275	ND	1.807	0.559
Count	894	934	325	34	766	930	ND	67	312	ND	4	72
p-value	<0.0001*			<0.0001*			<0.0001*			p<0.05*		
Analysis Method	one-way ANOVA			one-way ANOVA			t-test			t-test		

Statistical analysis comparing Acceptor ranges among A:D ratios per binned acceptor range. Each acceptor Range column (0-25, 26-50, 51-75, 76-100) includes statistical analysis among the A:D ratios (1:2, 1:1, 2:1). One-way ANOVA is used for analysis in binned acceptor ranges of 0-25, 26-50. t-test analysis is performed for binned acceptor ranges of 51-75 and 76-100. The results show that at a fixed range of acceptor intensities, E% is positively dependent on A:D ratios, as expected for intra-molecular FRET between Tfn molecules bound to homodimeric TfnR; inter-molecular FRET between clustered TfnR-Tfn complexes cannot be excluded (8). p-values < 0.05 are considered significant (*).

Supplementary Table S3 (for Figure 2A)

A:D ratio	T47D Short lifetime±SD (ps)	HMEC Short lifetime±SD (ps)
0:1	333±36	321±36
1:4	287±33	328±45
1:3	303±44	314±33
1:2	298±48	301±4
1:1	319±31	285±24
2:1	305±35	320±33
3:1	295±40	304±13

Short component lifetimes are estimated by biexponential fitting for both T47D and HMEC at A:D ratio from 0:1 to 3:1. The mean short lifetime for both T47 and HMEC is around 300ps. Small SDs are obtained(~32ps). These results indicate a high sensitivity and uniform detection despite the heterogeneity of donor intensities in the sample.

Supplementary Table S4 (Figure 2B)

Cell Type	T47D	HMEC
Slope	7.767 ± 0.5321	6.896 ± 0.5845
95% CI for Slope	6.290 to 9.244	5.274 to 8.519
r^2	0.9581	0.9530
p-value (slope comparison)	0.3029 (n.s.)	
analysis method	ANCOVA	

Statistical analysis of slopes of FD% vs. A:D ratios between T47D and HMECs. No statistical significance is detected between T47D and HMEC slopes using Ancova analysis, suggesting that TfnR-Tfn complexes show similar FRET behavior in HMEC vs. T47D cells, as shown in Supplementary Table 1. n.s. = not statistically significant (p>0.05).

Supplementary Table S5 (Figure 2C)

Acceptor Ranges (μg/mL)	2.5			5			10			20		
A:D ratios	1:2	2:1	3:1	1:2	2:1	3:1	1:2	2:1	3:1	1:2	2:1	3:1
FD%	14	25	40	14	24	32	14	25	31	14	25	31
SD	4	4	8	4	5	5	3	5	5	3	4	3
p-value	<0.001*			<0.01*			<0.01*			<0.001*		
Analysis Method	1-way ANOVA			1-way ANOVA			1-way ANOVA			1-way ANOVA		

Statistical analysis comparing % FD among A:D ratios using acceptor ranges of 2.5, 5, 10, and 20 μg/ml Tfn. p-value is the comparison of the % FD among all A:D ranges (1:2, 2:1, 3:1). The results show that at a fixed range of acceptor concentrations, FD% is positively dependent on A:D ratios, as expected for intra-molecular FRET between Tfn molecules bound to homodimeric TfnR; inter-molecular FRET between clustered TfnR-Tfn complexes cannot be excluded (8). p-values < 0.05 are considered significant (*).

Supplementary Table S6 (Figure 2D)

Cell Number	T47D ($1x10^5$ cells)	T47D ($1x10^4$ cells)
Slope	9.631 ± 0.7624	7.767 ± 0.5321
95% CI for Slope	7.514 to 11.75	6.290 to 9.244
r^2	0.9755	0.9816
p-value (slope comparison)	0.08 (n.s.)	
analysis method	ANCOVA	

Statistical analysis of slopes of FD% vs. A:D ratios in T47D cells at $1x10^5$ and $1x10^4$ cells. No statistical significance is detected using Ancova analysis, suggesting sensitivity of % FD is consistent across 1 $x10^5$to 1 $x10^4$T47D cells. n.s. = not statistically significant (p>0.05).

Supplementary Table S7 (for Figure 3A)

A:D ratios	20 µg/mL Short lifetime±SD (ps)	40 µg/mL Short lifetime±SD (ps)
0:1	333±35	322±42
2:1	320±38	336±18

Short component lifetimes measured in picoseconds are shown, indicating a high sensitivity and uniform detection despite the heterogeneity of donor intensities in the sample.

Supplementary Table S8 (for Figure 3B)

Amount of Tfn	20 µg/mL		40 µg/mL	
A:D ratios	0:1 (donor only)	2:1	0:1 (donor only)	2:1
FD%	11	39	13	37
SD	4	3	4	3
p-value	<0.001*		<0.005*	
Analysis Method	t-test		t-test	

Statistical analysis comparing % FD between 0:1 and 2:1 A:D ratios for each amount of Tfn (20 µg/ml and 40 µg/ml). Statistical significance is reached comparing 0:1 vs. 2:1 A:D ratios for all Tfn amounts. The results show that at a fixed range of acceptor concentrations, FD% is positively dependent on A:D ratios, as shown in supplementary table 5. p-values < 0.05 are considered significant (*).

Supplementary Table 9 (for Figure 3B)

A:D ratios	0:1 (donor only)		2:1	
Amount of Tfn	20 µg/mL	40 µg/mL	20 µg/mL	40 µg/mL
FD%	11	13	39	37
SD	4	3	3	3
p-value	0.5734 (n.s.)		0.4601 (n.s.)	
Analysis Method	t-test		t-test	

Statistical analysis comparing % FD between 20 µg/ml and 40 µg/ml at either 0:1 or 2:1 A:D ratios. No statistical significance between each comparison is detected, suggesting sensitivity of % FD is consistent across internalization of 20 µg/mL and 40 µg/mL of Tfn. n.s. = not statistically significant (p>0.05).

. **Supplementary Table S10 (for Figure 3B)**

Source of Variation	Interaction	A:D Ratio	Tfn amount
% Total Variation	0.56	94.77	0.00
p-value	0.3559 (n.s.)	< 0.0001*	1.0000 (n.s.)
Analysis Method	two-way ANOVA		

Two-way ANOVA table to determine which parameter, A:D ratio or Tfn amount accounts for the variation in results. As shown, Acceptor:Donor ratios demonstrate the strongest influence in variation of data. n.s. = not statistically significant (p>0.05); p-values < 0.05 are considered significant (*).

Supplementary Table S11 (for Figure 4A)

A:D ratios	Short lifetime±SD (ps)

0:1	318±34
1:2	317±50
1:1	306±44
2:1	322±39

Short component lifetimes are estimated by biexponential fitting for live mice at A:D ratio from 0:1 to 2:1. The mean short lifetime is around 300ps. Small standard deviations are obtained(~40ps). These *in vivo* results are consistent with the *in vitro* data and demonstrate that our imaging platform is sensitive and quantitatively matches *in vitro* results generated by confocal microscopy.

Supplementary Table S12 (for Figure 4B)

A:D ratios	1:2	2:1
FD%	24	36
SD	4	3
p-value	p< 0.0001*	
Analysis Method	t-test	

FD% values are listed for A:D ratios of 1:2 and 2:1. Analysis indicates statistical significance between low and high values supporting a linear increase in FD% as A:D ratios increase. p-values < 0.05 are considered significant (*).
